# Discovery of a bell-shaped dose response curve to melanin-concentrating hormone in the 3T3-L1 adipocyte model: low-dose MCH facilitates adipogenesis

**DOI:** 10.1080/21623945.2025.2594882

**Published:** 2025-11-27

**Authors:** Laurie B. Cook, Colin M. King, Hiba Y. Abdullah

**Affiliations:** Department of Biology, State University of New York at Brockport, Brockport, NY, USA

**Keywords:** Melanin-concentrating hormone, melanin-concentrating hormone receptor 1, adipogenesis, 3T3-L1, G protein-coupled receptor, lipid droplet

## Abstract

Melanin-concentrating hormone signalling pathways in the central nervous system are of significant clinical interest in treating appetite, sleep, and mood disorders. However, with the additional discovery of active MCH signalling pathways in peripheral tissues, knowing the degree to which cellular context influence MCH receptor function is increasingly important. In this study, we discovered MCH-mediated signalling responses that demonstrated bell-shaped dose response curves in multiple assays using both pre- and post-adipocyte 3T3-L1 models. MCH facilitated cell adhesion in pre-adipocytes, increased both the number and size of lipid droplets and inhibited lipolysis in adipocytes, with a maximum effective dose at 1 nM MCH. We hypothesize that the concentration of MCH cells are exposed to influences G protein bias at MCHR1 and/or signal switching to an unidentified pathway. Furthermore, this study elucidates the importance of hormone concentration when measuring GPCR signalling pathways in cell culture and tissue models.

## Introduction

Melanin-concentrating hormone (MCH) is a cyclic peptide that decreases energy expenditure [1], while also promoting appetite [2], addictive behaviours [3], REM sleep [4], memory storage and arousal [5], depression, and [6] anxiety [7] via signalling in the lateral hypothalamus of higher ordered mammals [8]. There has been considerable interest in elucidating the physiological processes under the control of MCH, and this has led to the additional discovery of peripheral tissues with MCH signalling pathways, including the pancreas [9], intestinal epithelia and the enteric nervous system [10] and adipose tissue [11]. Based on knock-out and pharmacological studies, MCH signalling pathway components are promising targets for obesity, sleep, and mood disorders [12].

MCH acts as an agonist at two different G protein-coupled receptors (GPCRs), MCHR1 and MCHR2, with the latter found in higher ordered mammals, including humans. *Mch* and *mchr1* knock-out mice are hyperactive and resistant to diet-induced obesity [13–15]; discoveries that initiated years of research into the targeted disruption of MCH signalling for clinical use in humans, however the mechanisms responsible for MCH-mediated actions are unclear and as an additional hurdle, most available MCHR1 antagonists that made it to clinical trials had off-target effects at the hERG channel, until only very recently [16,17].

Our current knowledge of MCH signalling has been obtained from primary adipocyte tissue, adipocyte cultures and neuronal-based *in vitro* and *in vivo* models, among others; most scientific focus has been to elucidate the effects of MCH on the central nervous system. Our lab has found that the murine 3T3-L1 adipocyte model, endogenously expressing MCHR1 [18], is excellent for studying the association of MCHR1 with primary cilia [19,20]; MCHR1 is one of a number of GPCRs known to compartmentalize to primary cilia in neurons [21–25]. Furthermore, in pre-adipocyte models, MCH can fuel leptin synthesis and secretion [11], promote cell migration [26], and facilitate gene expression changes for pathways involved in interleukin signalling, nuclear receptor signalling and circadian rhythm, and differentiation [20]. Researchers studying gene expression changes in epicardial adipose tissue (EAT) from patients with coronary artery disease found *mchr1* and *mchr2* were among the genes significantly upregulated compared to controls, and that adipocytes were significantly larger in EAT patients [27], suggesting that MCH signalling in organ-associated adipose tissue may have a significant biological role. Further elucidation of the signalling pathways for MCH and its receptors is critical for the advancement of this field.

MCH has been found to couple to G_i/o_ and G_q_ protein pathways in cell culture models, although the selectivity for each varies among cellular context; in CHO cells [28] and in HEK293 cells [29] MCH causes activation of ERK via both G proteins, but in SK-MEL37 melanoma cells MCHR1 only couples to G_i/o_ [30]. MCHR1 has many predicted post-translational modifications (reviewed in [31]) and it desensitizes via clathrin-mediated endocytosis in a β-arrestin-dependent internalization process, which GRK2 can facilitate [32]. Most early pharmacological studies on MCH signalling relied on heterologous, overexpression models like HEK293 cells [29,33,34]; CHO-K1 cells [28,35–37] and Xenopus oocytes [38]. Far fewer of these early studies utilized cell models endogenously expressing MCHR1, but some include models such as human peripheral blood mononuclear cells [39], SH-SY5Y neuroblastoma cells [40], SK-MEL37 melanoma cells [30] and murine 3T3-L1 pre-adipocytes [18]. One criticism of MCH signalling studies derived from these models is that the concentrations of MCH used to initiate MCHR1 signalling in them far exceeded physiologically relevant concentrations. Naufahu and colleagues (2017) reportecsma at picomolar to low nanomolar ranges [41]. Therefore, *in vitro* studies using 100 nM to 1μM hormone concentrations likely force artificial signalling responses that would never be achieved *in vivo*.

We report herein that MCH signalling in 3T3-L1 cells, both pre- and post-differentiation, exhibits a bell-shaped response that peaks at 1 nM across a wide range of tested dosages. Our results suggest that hormone treatment concentrations must be taken into consideration when profiling the pharmacology of MCHR1 and assessing physiological impact.

## Methods

### Cell culture

3T3-L1 pre-adipocytes *(ATCC, CL-173)* were maintained in 25 cm^2^ flasks in DMEM *(CellGro)* containing 10% bovine calf serum (BCS) (*Atlanta Biologicals*). When cells were 80% confluent, they were loosened with trypsin-EDTA, which was aspirated before cells detached from the substrate. Cells were then resuspended in media, seeded into two fresh flasks at a density of ~1:10, placed in a humidified tissue culture incubator at 37 degrees C with 5% CO_2_ and 95% humidity, fed every 3–4 days, and passaged when 80–90% confluent.

### Induction of 3T3-L1 adipogenesis

3T3-L1 pre-adipocytes were seeded into dishes and allowed to grow to confluency (Day −2) in DMEM +10% BCS. On Day 0, media was aspirated and differentiation media containing DMEM with 10% foetal bovine serum (FBS, *Atlanta Biological*), 10 μg/ml insulin (*SantaCruz*), 115 μg/ml isobutylmethylxanthine *(Acros)*, and 400 ng/ml dexamethasone (*AlfaAesar)* was added. Then, on Day 2, media was again replaced with DMEM with 10% FBS, but only 10 μg/ml insulin for two days. On Day 4, this media change was repeated, dropping the concentration of insulin to 250 ng/ml, and on Day 6 it was dropped again to 100 ng/ml. From Days 8–14, cells were cultured in DMEM +10% FBS without any additional additives.

### Plating efficiency assay

3T3-L1 pre-adipocytes were seeded into 12-well dishes in DMEM +10% BCS and immediately treated with MCH (*Peptides International)* or 10 μM isoproterenol was used as a positive control for lipolysis. Media and/or cells were recovered to a microfuge tube, gently pelleted and resuspended in 10 µl PBS. Ten microlitres of 0.4% Trypan Blue was added to label live cells and live versus dead cell counts and ratios were determined using a Countess Cell Counter (*ThermoFisher*).

### Cell adhesion assay

3T3-L1 pre-adipocytes ( < passage 7) were cultured in DMEM containing 10% BCS. The cells in 6-well dishes were incubated at 37°C in a 5% CO_2_ incubator. Cells were treated with MCH or PMC-3881-PI *(Peptides International)*, an MCHR1 antagonist, at the indicated concentrations over 6 days, with a media change occurring every other day. Media was aspirated, and cells rinsed for 3 min in PBS, then cells stained with 1% Crystal Violet for 10 min at room temperature. Following two tap water washes by dipping the plate in a large beaker, 1 ml of 1% SDS was used to solubilize the dye and an A570 was collected on a spectrophotometer.

### Oil red assay

Differentiated 3T3-L1 adipocytes in 24-well plates were fixed in 4% paraformaldehyde in PBS for 1 h, washed twice with 60% isopropanol, then air dried [42]. Oil Red stock solution was prepared by diluting 0.5 g Oil Red O to 100 ml with 98% isopropanol. Immediately before staining, the stock was diluted at a 3:2 ratio with water and filtered through Whatman paper. Five hundred microlitres of this working solution were added to each well and cells incubated at room temperature for 10 min. Following staining, the Oil Red was removed, and the cells were rinsed several times with distilled water, then air dried. Following image acquisition, Oil Red was solubilized with 250 μl 100% isopropanol, transferred to a 96-well plate and the A595 was recorded against a water blank using a Biotek Synergy H1 plate reader. Data was then exported to an MS Excel file for future analysis.

### Lipolysis assay

An AdipoSIGHT 3T3-L1 Lipolysis Assay Kit (*ZenBio)* was used for the quantification of both glycerol and non-esterified free fatty acids and both assays were performed according to manufacturer’s instructions. Glycerol and free fatty acid standard curves were used to ascertain exact micromolar concentrations of each in the extracellular media. All data points were collected in quadruplicate.

### Microscopy and image analysis

Phase contrast images were obtained soon after cell staining to reduce the loss of morphological detail using a Nikon Eclipse TE300 inverted microscope with a 40X objective and INFINITY3 Capture software. For quantification of lipid droplets, images of fifty randomly chosen adipocytes were captured, circles were drawn around each individual lipid droplet within the cell, and the number of circles within each cell were counted using the ‘analyze particles’ function of NIH ImageJ and exported to an MS Excel file. For cell size measurements, a straight line was drawn from one edge to the other of randomly chosen adipocytes.

## Results

### MCH facilitates plating efficiency of 3T3-L1 pre-adipocytes

**Figure 1.**
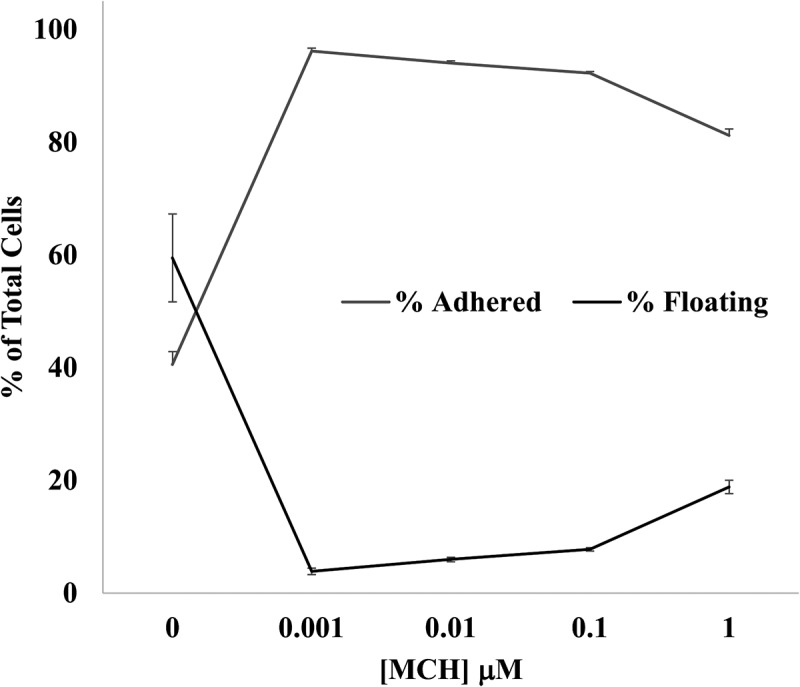
MCH improves 3T3-L1 plating efficiency. MCH was added in varying concentrations to fresh dishes in DMEM +10% BCS at the time of seeding, and cell concentrations were quantified 1 h after plating. Percent of total adhered and floating cells are graphed at each concentration of mch (*n* = 3, average ± sem is graphed). All MCH treatments showed statistical significance using a Student’s T-test against untreated cells at *p* < 0.05.

Adhesive interactions play critical roles in directing the migration, proliferation, and differentiation of cells. Since it has been shown that MCH has a transient effect on pre-adipocyte migration [26], we explored the dose-dependent effects of MCH on cell adhesion during plating with MCH concentrations up to 1μM. An et al. (2001) reported MCH-MCHR1 binding affinities at K_d_ values of 3.1 ± 0.4 nM [43], therefore we tested a broad range of MCH concentrations 1000-fold above and below the K_d_. MCH was added to fresh dishes at the time of seeding, and after 1 hour, we quantified the numbers of floating versus adhered cells. As you can see in Figure 1, MCH facilitated the plating efficiency of 3T3-L1 pre-adipocytes above control levels, with maximal effect at 1 nM MCH (*n* = 3 ± SEM, *p* < 0.05).

### Low dose MCH facilitates mitotic expansion of 3T3-L1 pre-adipocytes

**Figure 2.**
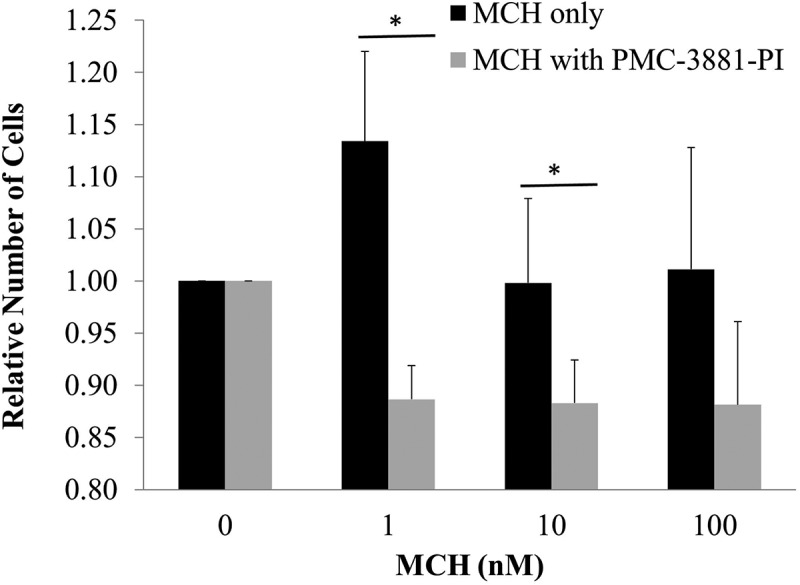
MCH improves 3T3-L1 pre-adipocyte viability in culture. 3T3-L1 preadipocytes were cultured for six days at the indicated doses in the presence (*n* = 3) or absence (*n* = 6) of 100 nM PMC-3881-PI, an MCHR1 antagonist. To assess cell viability, a crystal violet assay was performed and the A570 was recorded. Average ± sem is graphed. Statistical significance determined using a Student’s T-test. * indicates *p* < 0.05.

We next asked whether MCH treatment of pre-adipocyte cultures would trigger mitotic expansion of these cells, as is a characteristic of adipose tissue development [44]. We repeated our plating efficiency assay but increased our MCH exposure time to 6 days. Figure 2 illustrates that culturing pre-adipocytes with MCH post-plating facilitated cell survival for all concentrations tested, again with the most pronounced effect at the 1 nM MCH dose and progressively less of an effect over higher concentrations of MCH tested. This was completely blocked by simultaneous treatment with 100 nM PMC-3881-PI, an MCHR1 antagonist. Also, cell viability in the presence of antagonist was reduced over 6 days of treatment suggesting that MCHR1 harbours some basal level of agonist-independent signalling that facilitates cell survival.

#### Adipocyte size and lipid droplet number is sensitive to MCH

**Figure 3.**
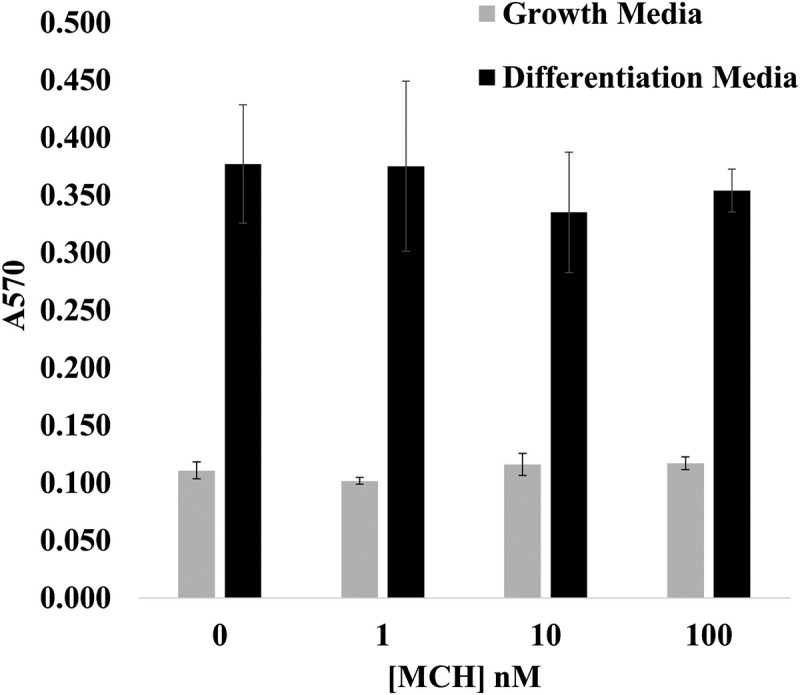
Oil red accumulation by 3T3-L1 adipocytes is unchanged by MCH. 3T3-L1 preadipocytes in 24-well plates were differentiated in the presence or absence of up to 100 nM mch. On the 10^th^ day of the differentiation protocol, cells were fixed and differentiated cells stained with Oil red, which was then eluted with 200 μl isopropanol and A570 measured. Ave ± sem is graphed, *n* = 3 replicates.

**Figure 4.**
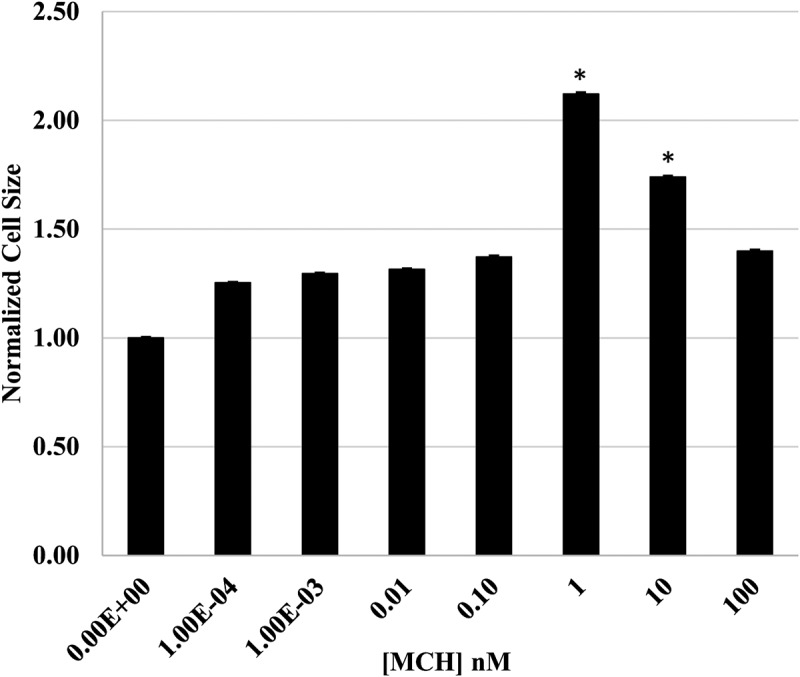
MCH promotes enlargement of adipocytes. 3T3-L1 preadipocytes were differentiated in the presence or absence of up to 100 nM mch. On the 10^th^ day of the differentiation protocol, cells were fixed and differentiated cells stained with Oil Red. Brightfield images were obtained on an inverted Nikon microscope using Infinity3 capture software. NIH ImageJ was used to measure the area of 30 individual cells and statistical significance determined using a Student’s T-test. * indicates *p* < 0.05, *n* = 30 replicates.

When we directly tested the effect of MCH treatment on 3T3-L1 adipocyte differentiation, we saw no significant effect in an Oil Red Assay as shown in Figure 3, which is consistent with the findings of Mul et al. (2013), who performed a similar dose response experiment *in vitro*. These same researchers also reported, however, that Pmch deficiency in rats reduced the size but not the number of adipocytes *in vivo* [45]. Since adipose tissue development involves both the mitotic expansion of pre-adipocytes and the accumulation of lipid droplets to accommodate long-term energy storage [46], we looked more deeply into the effects of MCH on 3T3-L1 adipocyte differentiation by quantifying adipocyte size following differentiation in the presence or absence of increasing concentrations of MCH. As you can see in Figure 4, when normalized to the area of control adipocytes, those treated with 1 nM MCH were 49% larger, whereas 10 nM and 100 nM treated adipocytes were 25% and 23% larger, respectively. Adipocytes receiving the 100 nM MCH treatment was not statistically different than untreated controls in this experiment.

**Figure 5.**
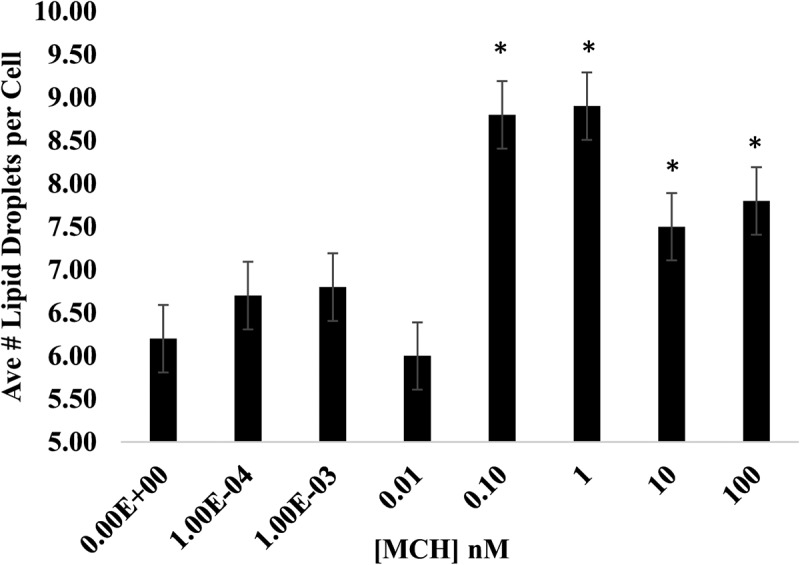
MCH increases the number of lipid droplets per cell. 3T3-L1 preadipocytes were differentiated in the presence or absence of up to 100 nM mch. on the 10^th^ day of the differentiation protocol, cells were fixed and differentiated cells stained with Oil Red. Brightfield images were captured on an inverted Nikon microscope using Infinity3 capture software. Droplets within 30 cells per treatment condition were quantified using nih ImageJ. Average number of droplets ±sem per cell is graphed. Statistical significance was determined using a Student’s T-test. * indicates *p* < 0.05, *n* = 30 replicates.

Adipogenesis is accompanied by the formation of lipid droplets within the cytoplasm serving as a mobile depot for triglyceride storage [47]. As a G_i/o_-coupled receptor, we did expect MCHR1 signalling to facilitate lipid droplet formation in a dose-dependent manner, however our initial attempts at detecting MCH-mediated changes in adipogenesis via Oil Red Assay revealed no detectable difference (Figure 3). When we analysed individual droplet numbers per cell, all concentrations at 0.1 nM MCH and above significantly increased droplet numbers, however the peak droplet numbers were found again at low dose MCH (0.1 and 1 nM concentrations), as shown in Figure 5.

#### MCH inhibits lipolysis by adipocytes

**Figure 6.**
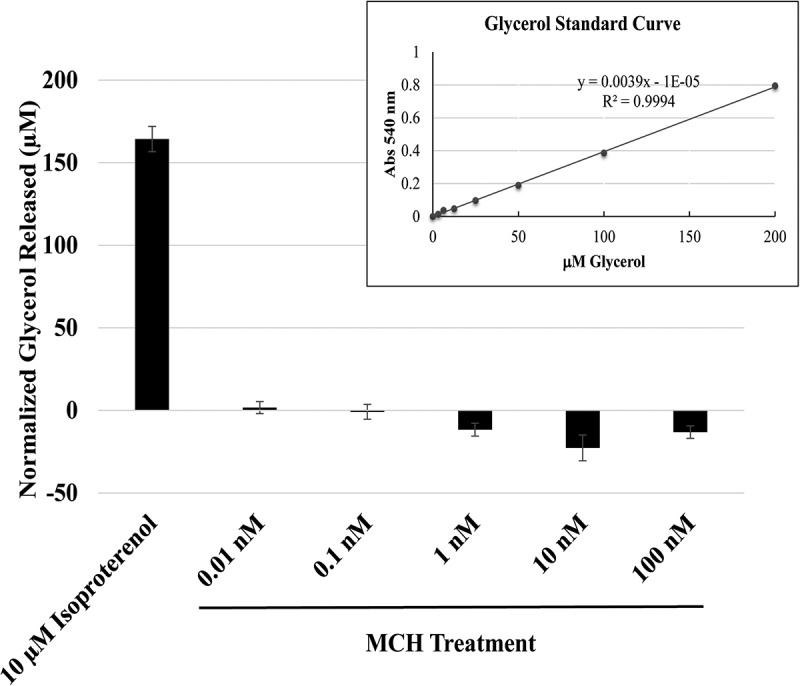
MCH treatment inhibits the release of glycerol from lipid droplets. 3T3-L1 adipocytes in 24-well dishes were treated with mch or isoproterenol (as a positive control for lipolysis) for 3 h. Glycerol released into the surrounding media was quantified. Standard curve R-value was 0.9994 as shown in the upper right corner. Data was normalized to vehicle control treatment. * indicates *p* < 0.05, *n* = 4 replicates.

**Figure 7.**
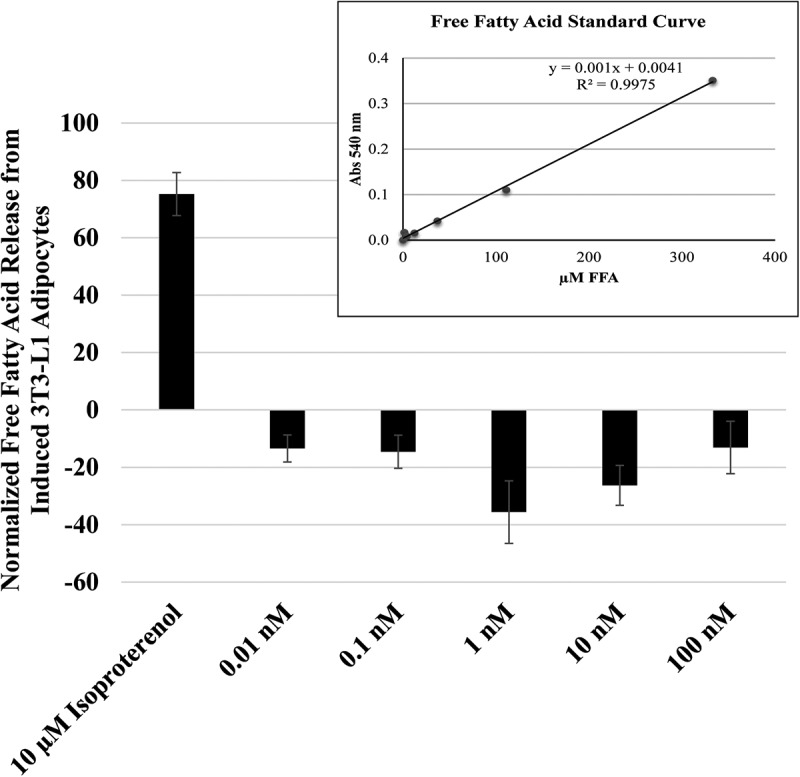
MCH treatment inhibits the release of free fatty acids from lipid droplets. 3T3-L1 adipocytes in 24-well dishes were treated with mch or isoproterenol (as a positive control for lipolysis) for 3 h. Free fatty acids released into the surrounding media was quantified. Standard curve R-value was 0.9975 as shown in the upper right corner. Data was normalized to vehicle control treatment. * indicates *p* < 0.05, *n* = 4 replicates.

Thus far, the evidence collected points to a role for MCH in facilitating lipid droplet integrity. As a G_i/o_-coupled receptor, MCHR1 is predicted to inhibit lipolysis. To directly test this, we measured the release of glycerol from 3T3-L1 adipocytes following a 3 h MCH treatment. As shown in Figure 6, our positive control, isoproterenol, predictably stimulated glycerol release and in Figure 7 free fatty acid release, from these cells. MCH treatments of 1 to 100 nM, however, reduced the release of glycerol (Figure 6) and free fatty acid (Figure 7) release below unstimulated levels, indicating that MCHR1 can suppress baseline lipolytic activity in 3T3-L1 adipocytes.

## Discussion

MCH signalling pathways are clinically relevant and could serve as a point of therapeutic intervention for sleep disorders, appetite, and mood. However, due to the presence of receptors for MCH in both central and peripheral tissues, any pharmacological intervention would necessitate an understanding of the potential significance of MCH signalling pathways throughout the body and within multiple cellular contexts.

Our study reveals an uncommon bell-shaped MCH dose response curve in un-ciliated pre-adipocytes and fully differentiated adipocytes using multiple assays. Sun et al. (2006) reported a similar finding in that β_2_-adrenergic receptor signalling in MEF cells is biphasic; at low nanomolar doses of isoproterenol, ERK was activated by a G protein-dependent mechanism, but at higher concentrations in the micromolar range, the activation was G protein-independent. Their work identified a now well-known G protein-independent pathway by β_2_-adrenergic receptor signalling to Src [48]. Another example of signal bias/switching has recently been described for the 5-HT_1a_ receptor activation of Gα_i3_, but not Gα_i2_. While they did use heterologous-expressing cell lines with high receptor concentrations for their studies, responses again functionally peaked in the nanomolar agonist concentration range before returning to baseline [49]. MCHR1 couples to both G_q_ and G_i/o_ signalling pathways in cell culture, but an interesting and yet unexplained potentiation of ERK signalling occurred when MCH-exposed, heterologous-expressing cells were given pertussis toxin (to block G_i/o_) and forskolin (to stabilize G_s_-produced cAMP) [29] suggesting an additional G protein-independent arm to the MCHR1 signalling scaffold exists. How compartmentalization of MCHR1 into primary cilia affects its signalling is still unknown.

An additional consideration is the role receptor desensitization might play in mediating the return of the dose–response curve to baseline for MCHR1. Bradley et al. (2002) demonstrated that MCHR1 mRNA levels are unchanged over the time course of 3T3-L1 cell differentiation, but downregulation of MCHR1 protein was detectable within 30 minutes of treatment with a high dose (1 uM) of MCH [18]. We previously demonstrated that GRK2 can facilitate MCHR1 internalization in a heterologous overexpression system [32], but there are no phosphorylation studies of MCHR1 using ^32^P-orthophosphate labelling, only functional mutagenesis studies [50]; agonist-induced recruitment of kinases to the intracellular portions of the receptor is also still hypothetical at this point. Kaya et al (2020) attributed a phosphorylation barcode in the intracellular regions of the D1 dopamine receptor to differences in G protein-coupling and downstream signalling [51], and these possibilities should be explored for the MCHR1 as well.

Ye et al. (2024) used cryo-EM to study structures of agonist (active) and antagonist-bound (inactive) MCHR1, finding small, subtle bi-modal structural transitions [52]. Simultaneously, He et al. (2024) obtained cryo-EM structures for the MCHR1-G_i_ complex, but the MCHR1 protein needed to be truncated by 69 amino acids, tethered to a fusion protein and overexpressed in insect cells to purify it effectively [12]. Neither study addresses the impact other MCHR1-binding proteins might have on agonist or G protein affinity for the receptor. A review by Kim et al. (2022) summarizes recent attempts at trapping GPCRs into specific signalling conformations to achieve a desired physiologic response. Signal bias has been studied extensively, for example, for α_2A_ adrenergic receptors and µ-opioid receptors, and it is estimated that at least 30% of GPCRs studied can ‘switch’ signals [53]. While we know MCHR1 has a slight affinity preference for G_i/o_ over G_q_ [12], the possibility that the local MCHR1 proteome, or localization of the receptor in primary cilia may influence that preference is as yet to be determined.

Our adhesion experiments revealed a low-dose MCH preference (1 nM) in promoting preadipocyte retention (Figure 1) and viability (Figure 2) during plating. In a previous study, we demonstrated that 1 µM MCH initiated an actin remodelling event that caused a rapid transient collapse in actin stress fibres, rounding up of the cell, and stimulation of cell migration over several hours [26]; however we never tested a broad range of MCH concentrations then as we did in the current study. A similar migration study was conducted in 3T3-L1 cells using the P2Y receptor, however agonist concentrations used in that experiment were also in the high micromolar range [54]. It has been suggested that pre-adipocytes can switch between proliferative and migratory phenotypes, with the latter associated more closely with an inflammatory state in human adipose tissue [55]. Our results suggest that any future models about how MCH influences adipose tissue differentiation and development should consider the dose at which hormone treatments are performed when drawing conclusions from the associated study. The data shown herein support the hypothesis that low dose MCH exposure facilitates the size (Figure 4) and number (Figure 5) of lipid droplets, potentiating the adipocyte phenotype. This is partially supportive of data published by others showing MCH increased the size but not number of adipocytes in rats [45]. Our data did show that droplet number was sensitive to all concentrations of MCH above 0.1 nM, however we detected a less dramatic effect at higher dosages of MCH (10 nM and 100 nM).

In conclusion, we identified a novel, bell-shaped dose response curve for MCH in the 3T3-L1 adipocyte model using multiple assays at different developmental stages of the cell. We postulate that the concentration of MCH cells are exposed to influences G protein bias at MCHR1 and/or signal switching to an unidentified pathway. We caution interpretation of previously published studies using supraphysiological concentrations of MCH to elicit signalling responses and encourage additional dose response studies in other model systems to elucidate receptor sensitivity and specificity, and elucidation of signal pathway preference for MCHR1.

## Data Availability

The data that support the findings of this study are publicly available in figshare at https://doi.org/10.6084/m9.figshare.c.7941842.
